# Radiation-Induced Tumor-Derived Extracellular Vesicles Combined with Tyrosine Kinase Inhibitors: An Effective and Safe Therapeutic Approach for Lung Adenocarcinoma with EGFR19Del

**DOI:** 10.3390/vaccines12121412

**Published:** 2024-12-14

**Authors:** Yao Li, Yaping Long, Xiangwei Ge, Pengfei Zhang, Tao Li, Liangliang Wu, Hao Fan, Zhijuan Du, Qiaowei Liu, Yi Hu

**Affiliations:** 1 Medical School of Chinese People’s Liberation Army (PLA), Beijing 100000, China; liyao1015359@163.com (Y.L.); gexiangwei301@163.com (X.G.); flowra7@126.com (P.Z.); drtaolee@outlook.com (T.L.); fanhaofanhao@163.com (H.F.);; 2 Department of Oncology, The Fifth Medical Center of Chinese PLA General Hospital, Beijing 100000, China; 15302157902@163.com; 3 Institute of Oncology, The First Medical Center of Chinese, PLA General Hospital, Beijing 100000, China; wuliang080228@163.com

**Keywords:** EVs, DC, radiation, NSCLC, EGFR

## Abstract

Background: Combining radiotherapy with targeted therapy benefits patients with advanced epidermal growth factor receptor-mutated non-small cell lung cancer (EGFRm NSCLC). However, the optimal strategy to combine EGFR tyrosine kinase inhibitors (TKIs) with radiotherapy for maximum efficacy and minimal toxicity is still uncertain. Notably, EVs, which serve as communication mediators among tumor cells, play a crucial role in the anti-tumor immune response. Methods To exploit the role of EVs in the delivery of tumor antigens, we formulated a therapeutic strategy that involves the use of radiation-induced tumor-derived EVs (TEXs) loaded onto dendritic cells (DCs) as a kind of vaccine in conjunction with EGFR TKIs and assessed the efficacy and safety of this approach in the treatment of EGFRm NSCLC. Results In our study, we characterized the release of immunogens as influenced by various modes of cell death, examining the impact of different levels of cell death under diverse irradiation modalities. Our results demonstrated that a radiation mode of 6Gy*3f exhibited the most promising potential to stimulate anti-tumor immune responses. This radiotherapy fraction, combined with TKIs, showed promising results in a tumor-bearing mouse model with an EGFR mutation, although there is a risk of radiation-associated pneumonitis. Furthermore, we found that 6Gy*3f-TEXs in vitro activate DCs and promote T cell proliferation as well as cytotoxic T lymphocyte-mediated tumor cell destruction. The administration of EGFR-TKIs combined DCs loaded with 6Gy*3f-TEXs exhibited the potential to inhibit tumor growth and mitigate the risk of pneumonitis. Together, the research shows that TEXs from high-dose fractionation radiation can mature DCs and boost the killing of cytotoxic T lymphocytes. Combining these DC vaccines with Osimertinib offers a promising and safe treatment for EGFRm NSCLC.

## 1. Introduction

Lung cancer continues to be the predominant cause of cancer-related mortality globally, with non-small cell lung cancer (NSCLC) comprising approximately 85% of all cases [1]. Among the subtypes of NSCLC, adenocarcinoma is the most common, frequently exhibiting distinct molecular alterations that promote tumor growth and progression [2]. A significant advancement in the targeted treatment of lung adenocarcinoma has been identifying and developing epidermal growth factor receptor tyrosine kinase inhibitors (EGFR-TKIs) [3]. EGFR-TKIs have markedly transformed the therapeutic paradigm for patients with EGFR-mutated NSCLC (EGFRm NSCLC) due to their high specificity and relatively favorable toxicity profile. Notably, Osimertinib, a third-generation EGFR-TKI, effective against both EGFR-sensitive mutations and T790M resistance mutations, has made substantial progress in the treatment of advanced lung adenocarcinoma, especially as a first-line therapy and in the management of brain metastases [4]. Despite the advantages of EGFR-TKIs, their efficacy in treating EGFRm-NSCLC remains constrained. Persistent oligo-residual lesions in advanced stages may lead to acquired drug resistance and an increased risk of distant metastasis [5].

Radiotherapy (RT) is a crucial component in the management of lung adenocarcinoma, particularly in achieving local control and in conjunction with chemotherapy and immunotherapy. It has long been recognized for its efficacy through mechanisms that induce DNA damage and subsequent cell death. More studies, however, have increasingly highlighted the role of RT in modulating both local and systemic immune responses to exert anti-tumor immune effects, including the promotion of antigen recognition of tumor-specific CD8+ T cells to attack both irradiated and non-irradiated tumors [6,7].Furthermore, the immunostimulatory impact of radiation is influenced by variable factors such as radiation doses and fractionation regimens [8,9]. Recently, there has been a growing interest in combining EGFR-TKIs with RT to enhance the anti-tumor activity and overcome resistance. Clinically, combining RT with EGFR TKIs may be more beneficial, as shown by the REFRACT study, which reported improved progression-free and overall survival, particularly in Asian patients with locally advanced EGFRm-NSCLC [10]. Nevertheless, pulmonary toxicity from combining RT and TKIs is concerning. The RECEL study reported that 16.7% of patients experienced severe radiation pneumonitis (RP) with RT and erlotinib. Another study found that 63.6% had moderate to severe RP with Osimertinib and thoracic radiotherapy [11]. More research on radiation patterns is needed to improve combination therapy and minimize side effects.

EVs are small vesicles released by cells that carry an extensive array of bioactive molecules, presenting promising applications in the therapeutic domain of lung cancer, including their use as targets for therapy, drug delivery vehicles, and EV-based immunotherapy. Tumor-derived EVs (TEXs) contain tumor-associated antigens (TAAs) and can transfer these to DCs, triggering antigen-specific T cell activation [12,13]. Radiation alters the quantity and makeup of TEXs, which interact with immune cells and modify the tumor microenvironment (TME), influencing tumor growth and treatment response [14]. Research has shown that irradiated NSCLC-derived EVs influence NK and B cell distribution in vitro, with effects varying by radiation dose and rate [15].

This study explored the optimal irradiation pattern to stimulate immune responses and examined the immune activation effects of EV-loaded DCs. The efficacy and superior lung safety of DCs loaded with radiation-induced TEXs (RA-TEXs) were validated in vivo in conjunction with TKIs in animals with an EGFRm background. The results offer a novel strategy for treating patients with advanced EGFRm NSCLC.

## 2. Materials and Methods

### 2.1. Construction and Processing of EGFR-19del Encoding LLC Cells

LLC (ATCC, Cat. CRL-1642) cell lines were purchased from Procell Life Science & Technology (Wuhan, China), cultured in DMEM with 10% FBS and 1% penicillin–streptomycin (Gibco, Grand Island, NY, USA), and authenticated via a short tandem repeat analysis. According to the literature [16], these cells were transfected with EGFR-Exon 19 Deletion (EGFR-19Del) overexpressing lentivirus from GeneChem (Shanghai, China) to express human EGFR-19Del protein. LLC cells were grown in six-well plates and infected with lentiviral particles. After 48 h, the medium was replaced with DMEM containing 2 µg/mL puromycin (Thermo, Waltham, MA, USA). Two weeks later, puromycin-resistant cells were collected and EGFR-19Del protein overexpression was confirmed via Western blot.

### 2.2. Apoptosis Detection

Cells were subjected to digestion using EDTA-free trypsin and subsequently gathered through centrifugation at 300× *g* for 5 min at 4 °C. Following a double wash with PBS, the cells were resuspended within a 100 µL buffer. Additionally, 5 µL FITC-Annexin V and 5 µL PI (DOJINDO, Kumamoto, Japan) were added and the cells were incubated for 5 min. After staining, 400 µL of buffer was gently mixed in. Apoptosis was quickly assessed using flow cytometry, identifying early apoptotic cells as Annexin V positive and late apoptotic or necrotic cells as Annexin V/PI double-positive.

### 2.3. ATP Release

The ATP release was quantified using an ATP detection kit (Beyotime, Shanghai, China). Cellular samples underwent treatment with a lysis buffer and subsequent centrifugation to isolate the supernatant. The resultant supernatant was combined with an ATP working solution within the detection wells and homogenized. The value of RLU was measured using a luminometer. The ATP concentration was determined by converting the RLU values through a standard curve derived from the standard samples.

### 2.4. Bone Marrow-Derived Dendritic Cell (BMDC) Induction and Culture

Femoral bones from euthanized mice were used to extract bone marrow cells, which were then filtered and lysed to remove Red Blood Cells. The cells were cultured in RPMI Medium 1640 with 10% FBS, 1% penicillin–streptomycin, 20 ng/mL IL-4, and 20 ng/mL GM-CSF (PeproTech, Rocky Hill, NJ, USA). After 72 h, non-adherent cells were discarded and adherent cells were cultured, with half the medium being replaced every two days. On the ninth day, immature DCs (iDCs) were induced and differentiated. Semi-adherent and suspension cells were collected, cleaned, and prepped for further experiments. The cells were incubated with FITC-CD11c (Biolegend, San Diego, CA, USA) for 30 min in the dark for staining. The purity of these cells was then analyzed using flow cytometry.

### 2.5. EV Isolation and Characterization

Cells demonstrating successful expression of EGFR-19Del protein were required to be substituted with a serum-free medium (Yeasen, Shanghai, China) to extract EVs after reaching the appropriate density. These cells were irradiated using an RS2000 Irradiator with parameters of 160 Kv and 25 mA and a dose rate of 1.175 Gy/min. EVs were extracted from LLC-EGFR19Del cell culture supernatants. Initially, cells and large particles were removed by centrifuging at 300× *g* for 10 min. Crude EVs precipitates were then obtained by centrifuging at 2000× *g* for 30 min. Further centrifugation at 10,000× *g* for 60 min was performed to eliminate shed microvesicles. The supernatant was then ultracentrifuged at 100,000× *g* for 120 min at 4 °C in an ultracentrifuge (Beckman Optima XPN-100, Beckman Coulter, FL, USA). The precipitate was resuspended with PBS and the ultracentrifugation was repeated once. After resuspension with sterile PBS, the precipitate was stored in portions at −80 °C. The EVs were analyzed for particle size using a Flow NanoAnalyzer (NanoFCM Inc., Xiamen, China) and for morphology using transmission electron microscopy. Post-protein cleavage, the supernatant was centrifuged and the extracted proteins were stored and used for detection.

### 2.6. Analysis of DCs Activation Induced by EVs

Mouse iDCs were procured as delineated in previous studies. Subsequently, these cells were cultured for 24 h by incorporating irradiated or non-irradiated tumor-derived EVs (20 µg/mL) or Lipopolysaccharides (LPS) at a concentration of 1 ng/mL (Sigma, St Louis, MO, USA). Following incubation, the cells were harvested and prepared for flow staining to identify the DC phenotype. Concurrently, the supernatants were also collected to quantify the cytokines secreted by the DCs.

### 2.7. T Cell Proliferation and Tumor-Specific CTL Response In Vitro

Mice spleens were processed to isolate lymphocytes (Solarbio, Beijing, China). T lymphocyte concentration was determined via flow cytometry. These T cells were activated with CD3, CD28, and IL-2 (Biolegend, San Diego, CA, USA) and stained with 5 µM Carboxyfluorescein Diacetate Succinimidyl Ester (CFSE) (Thermo, Waltham, MA, USA) for 10 min at 37 °C, then washed with a 10% FBS medium. Subsequently, these CFSE-labeled T cells were co-incubated with DCs treated with EVs from various treatments. After 3 days, T cell-secreted cytokines were measured using ELISA and the expression of CFSE of T cells was also detected.

T cells activated by DCs functioned as effector cells/E, interacting with the unlabeled LLC-EGFR19Del as target cells/T, and these cells were co-incubated at the E/T ratios (5:1, 10:1, and 20:1) for 24 h. The CTLs’ killing activity was evaluated by measuring Lactate dehydrogenase (LDH) release and Annexin V-positive expression in flow cytometry. The LDH release kit (Beyotime, Shanghai, China) was used to determine the 450 nm absorbance value and cytotoxicity (%) = (absorbance of treated sample − absorbance of control sample)/(absorbance of maximum enzyme activity in sample − absorbance of control sample) × 100%. Annexin V staining was used to analyze the percentage of apoptotic cells.

### 2.8. Animal Model and Group Treatment

Six-week-old female C57BL/6 mice were injected with 1.0 × 10^6^ LLC-EGFR19Del and divided into groups randomly, each comprising five individuals when tumors reached an average size of 100 mm^3^. The treatment regimens administered were as follows: (1) Osimertinib, delivered via oral gavage, suspended in 0.5% CMC-Na at a dosage of 50 mg/kg per day for two weeks. (2) A daily dose of 6 Gy radiation targeted at the tumor for three consecutive days post-grouping utilizing the same radiation apparatus as previously described. (3) Intravenous administration of DCs stimulated with EVs (20 µg/mL) was performed every three days at a dosage of 1 × 10^6^ cells per 100 µL. (4) The combined treatment with Osimertinib and RT/DCs. Tumor dimensions were measured bi-dimensionally every three days using calipers, and tumor volume was calculated using the formula of volume (mm^3^) = (width)^2^ × (length) × 1/2 to monitor tumor growth. Additionally, animal weights were recorded every three days.

### 2.9. Animals Sampling

After completion of treatment, the ocular tissues were removed to collect peripheral blood under anesthesia. The serum was collected by resting for 30 min and centrifuged at 2000× *g* for 20 min. The blood sample for flow staining was lysed to remove erythrocytes, followed by washing and antibody staining. Organs like the heart, liver, spleen, and lungs were removed and preserved in paraformaldehyde; then, HE was stained for safety evaluation. The spleens were extracted from the mice, macerated on a 200-mesh sieve, gently crushed, and rinsed with buffer. The homogenate was centrifuged at 300× *g* for 5 min, and cells were washed with PBS after lysing erythrocyte. For cancer-tissue processing, about 300 mg of tissue was minced and mixed with a digestive solution, namely Collagenase Type IV and DNase I (Sigma, St Louis, MO, USA), then incubated at 37 °C for 35 min with shaking. The mixture was rinsed with PBS and filtered through a 70 μm cell strainer (Biosharp, Hefei, China) with 1%–2% fetal bovine serum. The filtrate was subsequently centrifuged and resuspended in preparation for staining.

### 2.10. Flow Cytometry Detection

After adjusting the cell concentration, the Fc segment of the cell suspensions was blocked. T cells were stained with PE-CD45/BV421-CD45, APC-CY7-CD3, PE-CY5-CD4, PE-CY7-CD8, BV510-CD25, BV421-Foxp3 markers. DCs were marked with FITC-CD11c, PE-CY7-CD40, APC-CD80, BV421-CD86, and PERCP-MHC-II. Macrophages were labeled with PE-CY7-CD11b, FITC-F480, and APC-CD206. All of the antibodies were obtained from Biolegend, CA, USA. Surface markers were stained under light-protected conditions for 30 min, followed by a washing step and subsequent detection. Staining of intracellular markers (Foxp3) was conducted post-fixation, and permeabilization was conducted utilizing the FIX/PERM kit (Biolegend, CA, USA). The system was resuspended with 200 µL volume to use the flow cytometer BD FACSCantoII (BD Biosciences, Milpitas, CA, USA) for detection. The data were analyzed statistically by FLOW Jo 10.6.

### 2.11. Immunohistochemical Staining and Analysis

The tissue samples underwent dehydration, embedding, sectioning, xylene, alcohol treatment, and microwaving for antigen repair. They were then treated with 3% H_2_O_2_ to remove peroxidase, sealed with 0.5% BSA, and incubated with anti-KI67 antibody- ab15580 (Abcam, Shanghai, China) at a concentration of 0.5 µg/mL overnight, followed by secondary antibody treatment and DAB colorimetric observation. Immunohistochemical images were semi-quantitatively analyzed using Image J 1.8.0.

### 2.12. ELISA Analysis

The quantification of HMGB1, IFN-γ, IL-12, IL-10, IL-4, and TNF-α expressions was conducted utilizing Elisa kits (Boster Company, Wuhan, China). Per the guidelines, cell culture supernatant or serum samples were amalgamated with the solid well-coated antibody at 37 °C. After the washing process, the enzyme-labeled antibody was incorporated into the system. Thereafter, a colorimetric reaction was induced by the substrate within 10 min and the value of O.D. was ascertained at 450 nm utilizing an enzyme marker.

### 2.13. Western Blot Analysis

Cells were lysed on ice with a lysis solution, protease inhibitor, and phosphorylase inhibitor for 30 min and mixed with a loading buffer. After boiling for 20 min, the protein was quantified using a BCA kit. Equal protein amounts (50 μg) were separated on 12% SDS-PAGE and transferred to a PVDF membrane. The membrane was blocked with 5% skimmed milk for 1 h at room temperature, then incubated with mouse antibodies of HSP70, TSG101, anti-Alix, anti-Calnexin, LC3II/I, P62, GAPDH, and anti-human EGFR (E746-A750del Specific) with a dilution ratio of 1:1000 (Cell Signaling Technology, Danvers, MA, USA) at 4 °C overnight. After incubation for 1 h with HRP-conjugated goat anti-mouse secondary antibody (Cell Signaling Technology, MA, USA), proteins were detected using ECL (Thermo Fisher Scientific, MA, USA) and the strip images were analyzed with Image J for statistical evaluation.

### 2.14. Data Analysis

Data values from in vivo and in vitro experiments were recorded as mean ± standard deviation. Differences between two groups and between three or more groups were compared using unpaired *t*-tests and one-way ANOVA in GraphPad Prism 10 software, respectively. *p* < 0.05 indicates a statistically significant difference.

## 3. Results

### 3.1. Radiation Dose-Dependently Promoted Apoptosis, Autophagy, and Immunogenic Cell Death (ICD) of Tumor Cells and the Radiation Fraction Matters

**Figure 1:**
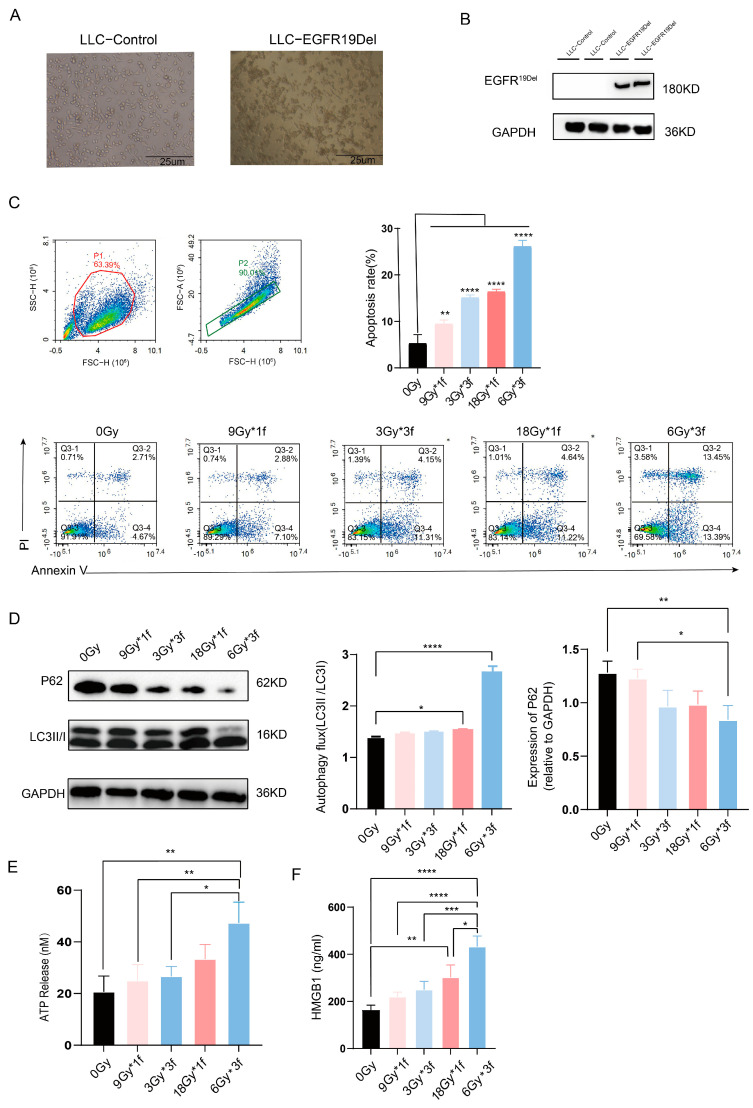
Radiation dose and fraction-dependently promoted apoptosis, autophagy, and ICD of tumor cells. (**A**). Images of cells were taken to detect the lentiviral or control group. (**B**). The protein expression level of EGFR19Del was detected by Western blot. (**C**). Apoptotic manifestations of tumor cells after different treatments were detected by Annexin V/PI flow cytometry. (**D**). Proteins associated with autophagy were validated and analyzed by Western blot. (**E**). The level of ATP release by cells was measured through chemiluminescence detection. (**F**). The content of HMGB1 in the supernatant was detected by ELISA analysis. * *p* < 0.05, ** *p* < 0.01, *** *p* < 0.001, **** *p* < 0.0001.

To determine the transfection efficiency of the EGFR19Del virus, cells of both the control and EGFR19Del-lentivirus transfected underwent puromycin screening and protein assays after 72 h of infection. The control group cells all died, while the EGFR19Del-lentivirus group cells grew well under the microscope. Western blot assays also confirmed the successful construction of LLC-EGFR19Del (Figure 1A,B). LLC-EGFR19Del, irradiated with X-rays, showed swelling, nuclear consolidation, unclear morphology, and increased fragmentation after 48 h (Figure S1). Research indicates that tumor cell apoptosis and autophagy can activate anti-tumor immune cells by releasing ATP and enhancing antigen presentation, respectively [17,18]. Thus, we first used apoptosis and autophagy to assess the tumor immune response to different irradiation patterns, examining four radiation doses’ effects on tumor cell death. Results showed higher doses increased apoptosis and autophagy. All irradiated groups had significant apoptosis compared to the unirradiated group, with the 6Gy*3f dose being the most effective (Figure 1C). Radiation at 3Gy*3f and 18Gy enhanced autophagy compared to the unirradiated group, and 6Gy*3f had a statistically significant change. With the same total dose, segmented radiation (6Gy*3f) induced more autophagy and apoptosis than single doses (18Gy*f) (Figure 1D). For the primary assessment of ICD, the ELISA for HMGB1 in the supernatant and measurement of ATP release revealed that the two groups with the highest radiation dose exhibited an active process, which increased the secretion of HMGB1 and ATP in the supernatant. Compared with other groups, 6Gy*3f resulted in a marked increase in the release of ATP and HMGB1. Overall, these findings hint that 6Gy*3f showed the highest potential for inducing immunogenic cell death.

### 3.2. Radiation Therapy Optimizes the Efficacy of TKIs and Increases CD8^+^ T Cell Infiltration in the TME but Is Associated with a Potential Risk of Radiation Lung Damage

**Figure 2:**
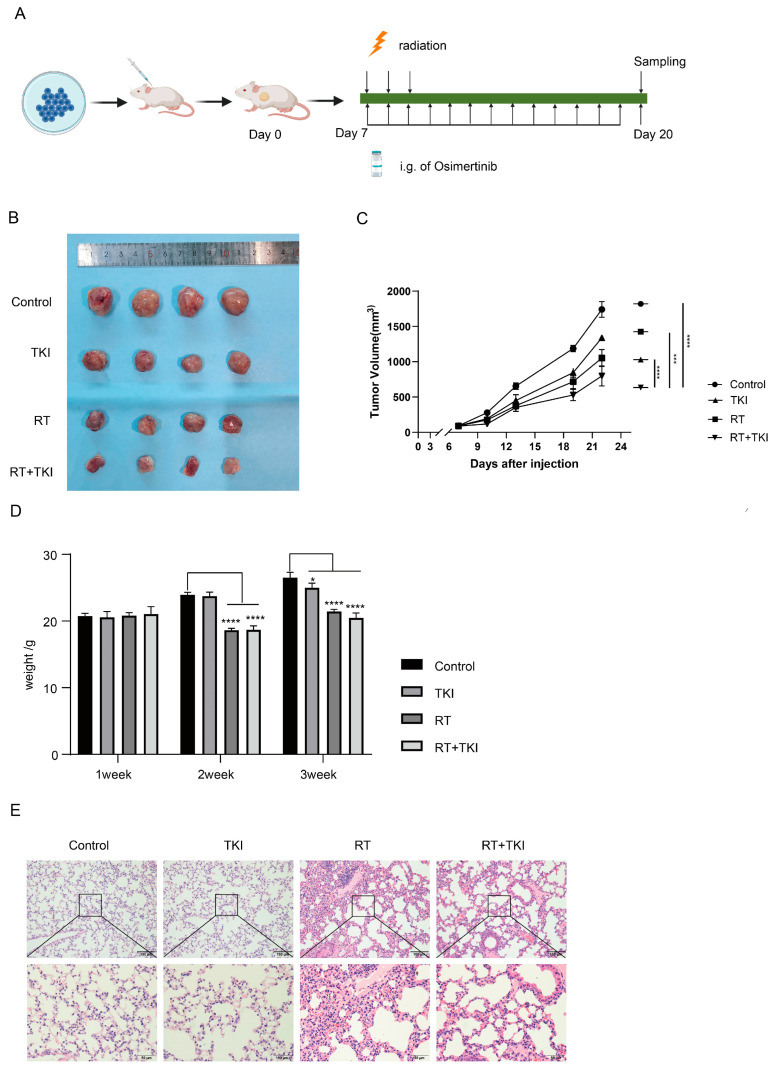

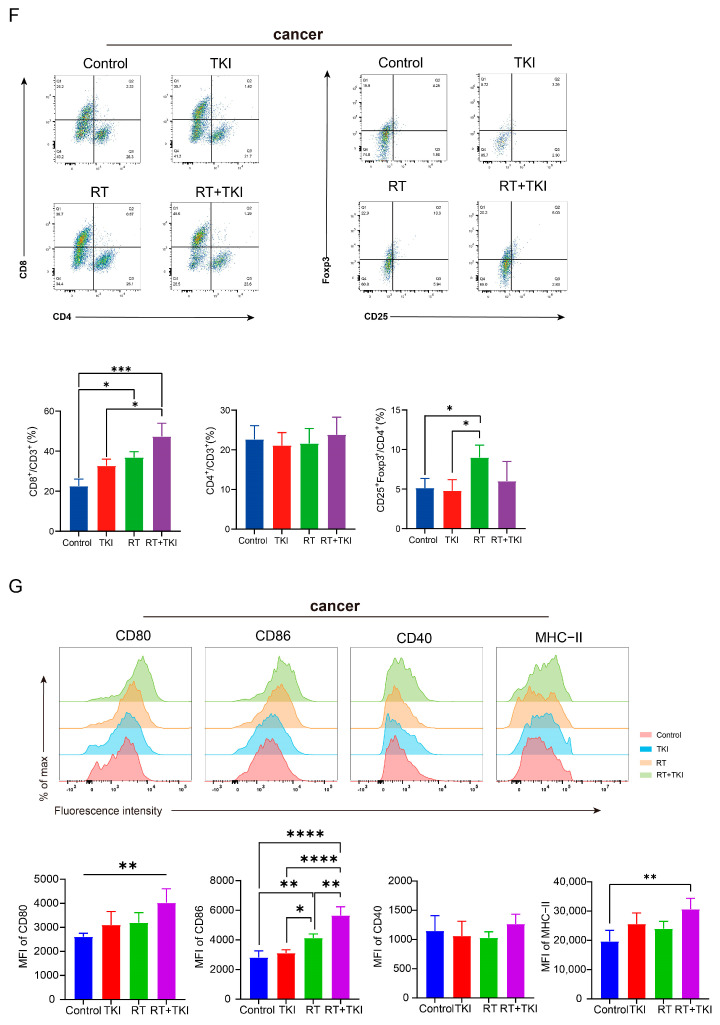

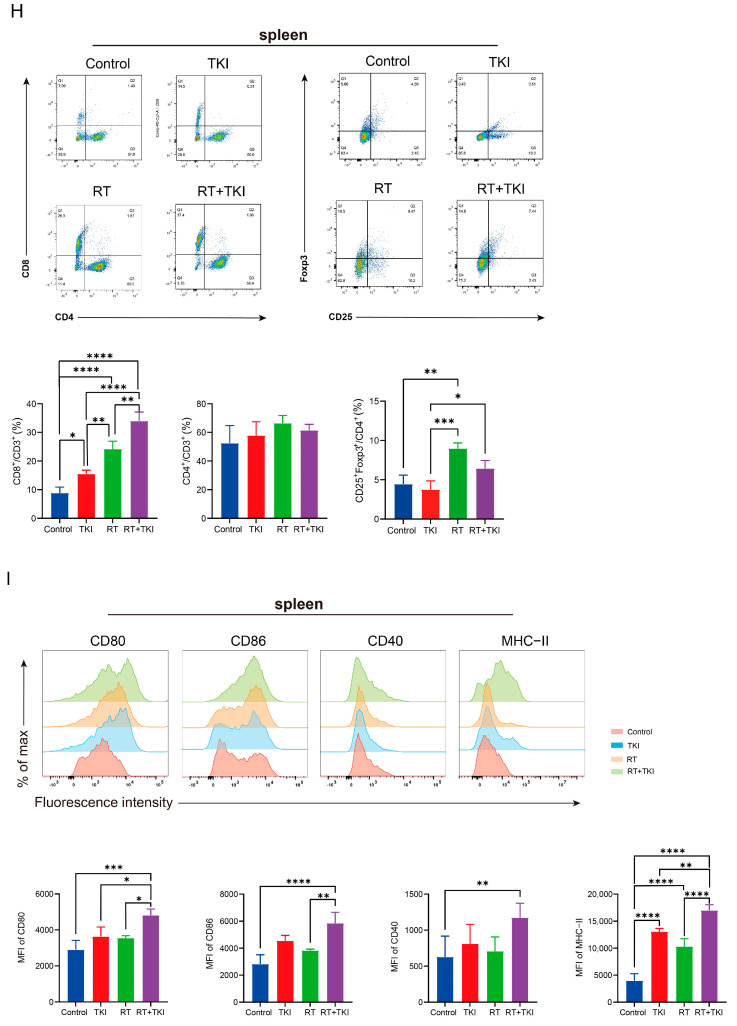
RT enhanced the efficacy of TKIs with increased CD8 T cell infiltration of the TME and risk of pneumonia. (**A**). Groups of four mice were inoculated subcutaneously with 1 × 10^6^ LLC-EGFR19Del cells on day 0. On the seventh day, three consecutive 6Gy irradiation sessions were administered once daily. Gavage with Osimertinib was given to mice every day from day 7 to day 19. The mice were sacrificed on day 20 to evaluate the immune response. (**B**,**C**). Photograph and volumes of tumor tissues from mice. (**D**). The body weights of mice in each group were measured every week. (**E**). Sections from the lung were observed by HE staining. (**F**,**H**). The tumors or spleens of C57 mice were stained using anti-mouse CD45, CD3, CD8, CD4, CD25, or Foxp3 and analyzed using flow cytometry to detect CD4, CD8, and Tregs. (**G**,**I**). DCs from tumor or the spleen were gated as CD45^+^ CD11c^+^ cells and analyzed to evaluate the expression of MHC-II, CD40, CD80, and CD86. The Schema diagram of Figure 2A was created with BioRender.com. RT: radiation therapy, TKIs: tyrosine kinase inhibitors, TME: tumor microenvironment. * *p* < 0.05, ** *p* < 0.01, *** *p* < 0.001, **** *p* < 0.0001.

Local treatments like radiotherapy are essential for stage IIIB EGFR mutation-NSCLC patients, but combining EGFR-TKIs with radiation may worsen lung injury due to limited available data [19,20,21]. In our experiment, we injected LLC-EGFR19Del cells into C57 mice to create a tumor model (Figure 2A). Tumor volume measurements showed that the combined treatment group had the most significant tumor reduction, as both TKI and radiation therapy effectively slowed tumor growth compared to the control group (Figure 2B,C). To ensure safety, we regularly monitored the mice’s body weights and performed HE staining on their internal organs. The data showed that mice undergoing RT experienced significant weight loss post-radiation, with slow recovery starting around day ten (Figure 2D). The lung tissues in both the control and TKI groups showed normal structures with intact alveolar intervals and well-expanded lumens. In contrast, the irradiated groups displayed mild alveolar wall thickening, edema, and increased gelatinous secretion, indicating congestion and inflammatory cell infiltration that is typical of pneumonitis (Figure 2E). The staining of other organs revealed no clear pathological abnormalities (Figure S2). In summary, while combination therapy offers clear anti-tumor benefits, it also poses a risk of lung injury from radiation.

In addition, we conducted an extensive examination of various immune cell populations within the murine model. The treatment groups, especially those receiving RT, exhibited a marked increase in CD8^+^ T cell infiltration within the TME relative to the control group. However, radiation did not notably impede the proportion of regulatory T cells (Tregs). This might be due to the hypoxic environment and increased release of factors like TGF-β and IL-1β, which promote Tregs expansion post-radiation therapy. The TKI group improved the activity of tumor-infiltrating T cells. The combination groups exhibited a marked increase in the infiltration of CD8^+^ T cells compared to other groups, and this enhancement in anti-tumor immune activity was observed without any exacerbation of immunosuppressive-Treg differentiation compared with RT (Figure 2F). Immune cells from the spleen and blood were examined to evaluate immunological activity. A flow cytometry analysis showed that TKI and RT treatment consistently increased specific cytotoxic CD8^+^ T cells and the combination therapy significantly mitigated the propensity for RT-induced TREG infiltration in the spleen, consistently highlighting the advantages of the synergistic approach (Figure 2H and Figure S3).

Due to conflicting reports on DCs activity in the TME after radiation [22,23], we examined DCs maturation markers in tumors and spleens. Our results showed the highest percentage of mature DCs in the combination treatment group (Figure S4) along with increased CD86, CD80, and MHC-II in tumor-infiltrating DCs compared with the control. (Figure 2G). Similarly, compared to the control group, the spleen had more dendritic cells (Figure S4), especially the cDC1 subset (CD11c^+^). The primary effect of treatment with TKI or RT was an upregulation of MHC-II. Importantly, the combined therapy group exhibited elevated expression levels of CD80, CD86, CD40, and MHC-II, suggesting a more robust DC-related immune response (Figure 2I).

Research shows that RT and EGFR TKIs affect extracellular ATP, which can enhance macrophage pro-inflammatory behavior and tumor antigen presentation [24]. However, our study found that treatment in three groups did not reduce tumor/spleen-associated macrophage infiltration or change the M1/M2 ratio compared to the control group (Figures S5 and S6).

### 3.3. Identification of EVs Secreted by Tumor Cells

**Figure 3:**
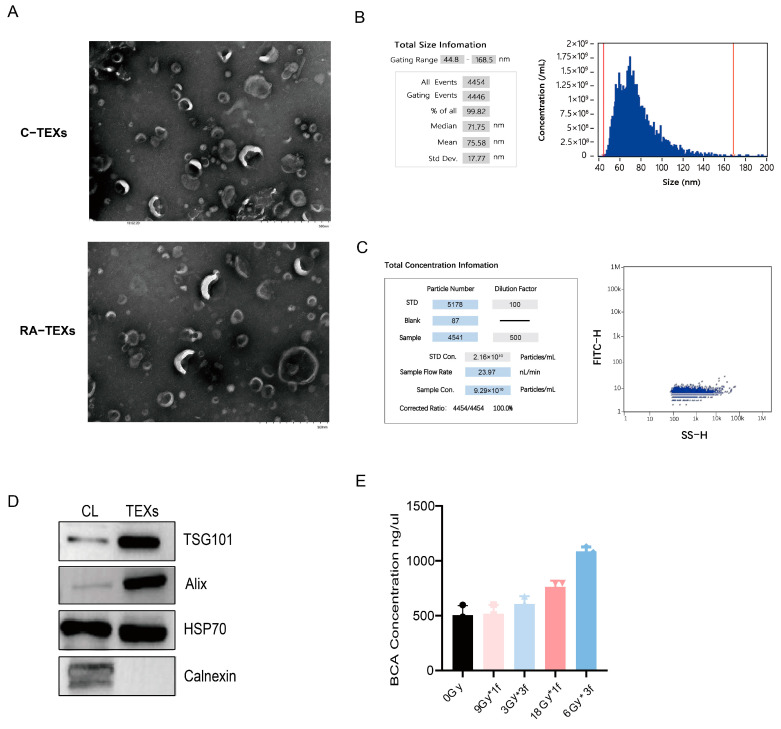
Characterization of EVs. (**A**). EV morphology revealed via transmission electron microscopy. (**B**,**C**). Particle size distribution in C-TEXs measured via a nanoparticle-tracking analysis. (**D**). Western blot analysis of EV-specific surface markers of C-TEXs. (**E**). Detection of exosomal protein secreted by tumor cells after different radiation by BCA assay.

After radiation, cells were given a serum-free EVs-specific medium and EVs were extracted via ultracentrifugation. TME images showed that RA-TEXs and unirradiated tumor-derived EVs (C-TEXs) had similar morphologies and sizes, resembling cup and saucer vesicles with diameters of 40–170 nm in a bilayered membrane structure (Figure 3A). Nano FCM analysis indicated an average EV particle size of 71.75 nm, with 99.82% of particles ranging from 44.8 to 168.5 nm in C-TEXs (Figure 3B,C). Protein extraction revealed the presence of the EV markers Alix, HSP70, and TSG101, but not Calnexin. A cell lysate (CL) was used as a control. The results confirmed that the cell supernatant extracts were EVs (Figure 3D). Furthermore, our findings show that RA-TEX protein levels were significantly higher per 100 mL of cell culture supernatant after the same cell-seeding operation compared to C-TEXs. Radiation doses of 6Gy*3f and 18Gy×1f notably increased protein content even though it is not certain whether the increase is due to the secretion of more EV particles or radiation stimulating the inclusion of more proteins within EVs (Figure 3E).

### 3.4. Radiation-Induced EVs Promote the Expression of Surface Co-Stimulatory Molecules and Cytokine Secretion During BMDC Maturation

**Figure 4:**
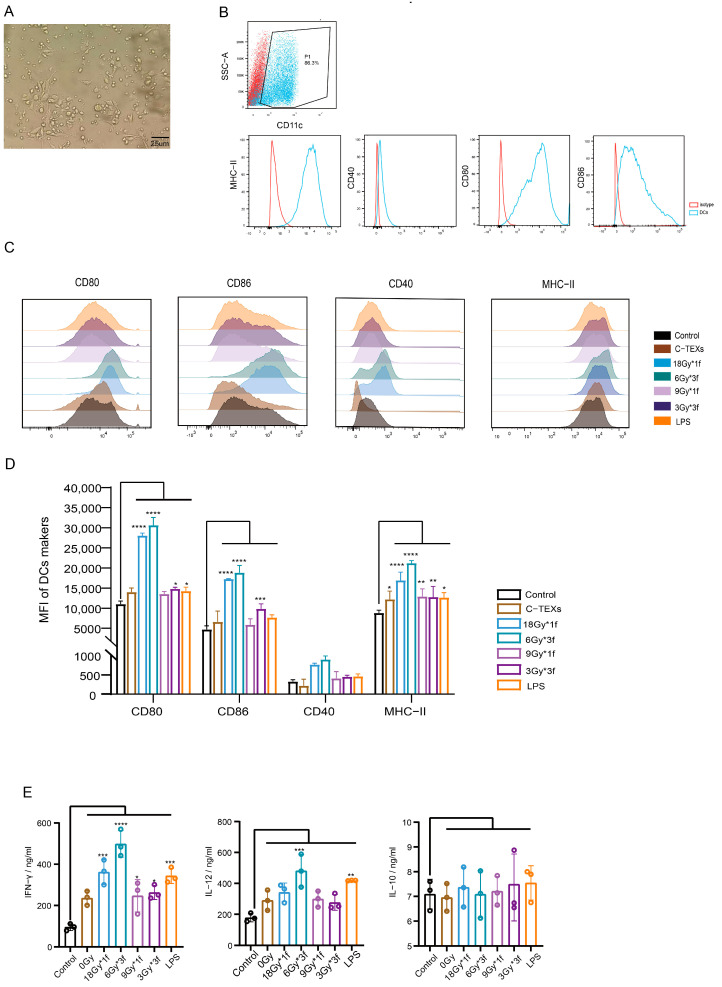
Radiation-induced EVs promoted BMDC maturation. (**A**). Typical DC morphology on day 9. (Magnification 40×). (**B**). BMDCs were positive for the surface molecules of CD11c, MHC-II, CD40, CD80, and CD86. (**C**,**D**). The expression of co-stimulatory molecules of BMDCs after 24 h of EVs or LPS treatment in each group was detected by flow cytometry. (**E**). The cytokine production of IFN-γ (the left), IL-12 (the middle), and IL-10 (the right) were analyzed by ELISA. * *p* < 0.05, ** *p* < 0.01, *** *p* < 0.001, **** *p* < 0.0001 .

The BMDC culture followed the protocol from a previous study [16]. After 9 days of induction, the cells increased in size and displayed dendritic protrusions. Successful differentiation was confirmed, with over 85% of cells testing positive for CD11c, and basic BMDC surface molecule expression was detected (Figure 4A,B).

TEXs contain TAAs and immune-related proteins that activate CD8^+^ T lymphocytes via DCs, resulting in strong anti-tumor responses [25]. In the study, we analyzed the expression of co-stimulatory molecules on the surface of BMDC following a 24 h treatment with EVs or LPS. The findings revealed that LPS slightly increased CD80 and MHC-II expression and RA-TEXs enhanced DC maturation in a radiation dose-dependent manner, with 6Gy*3f-TEXs being the most effective, which doubled CD80, CD86, and MHC-II levels relative to controls, indicating more mature DCs with an improved antigen-presenting capacity (Figure 4C,D).

In addition to upregulating DCs maturation markers, TEXs stimulate cytokine production. Our research found that RA-TEXs, especially 6Gy*3f-TEXs, significantly increased the expression of IFN-γ and IL-12, which are essential for the differentiation of T helper cell 1. There were no noticeable variations in IL-10 expression, inducing immune tolerance (Figure 4E). LPS and C-TEXs showed only a slight increase in pro-inflammatory factor production. Previous studies indicate that non-specific or insufficient absorption of soluble antigens fail to fully stimulate DC maturation [26]. We hypothesize that RA-TEXs are more effective in promoting DC maturation due to TAA-specificity. In contrast, specific radiation patterns that expose antigens may improve DCs’ antigen uptake.

### 3.5. Radiation-Induced EVs Enhance the Ability of DCs to Promote T Lymphocyte Proliferation and Killing In Vitro

**Figure 5:**
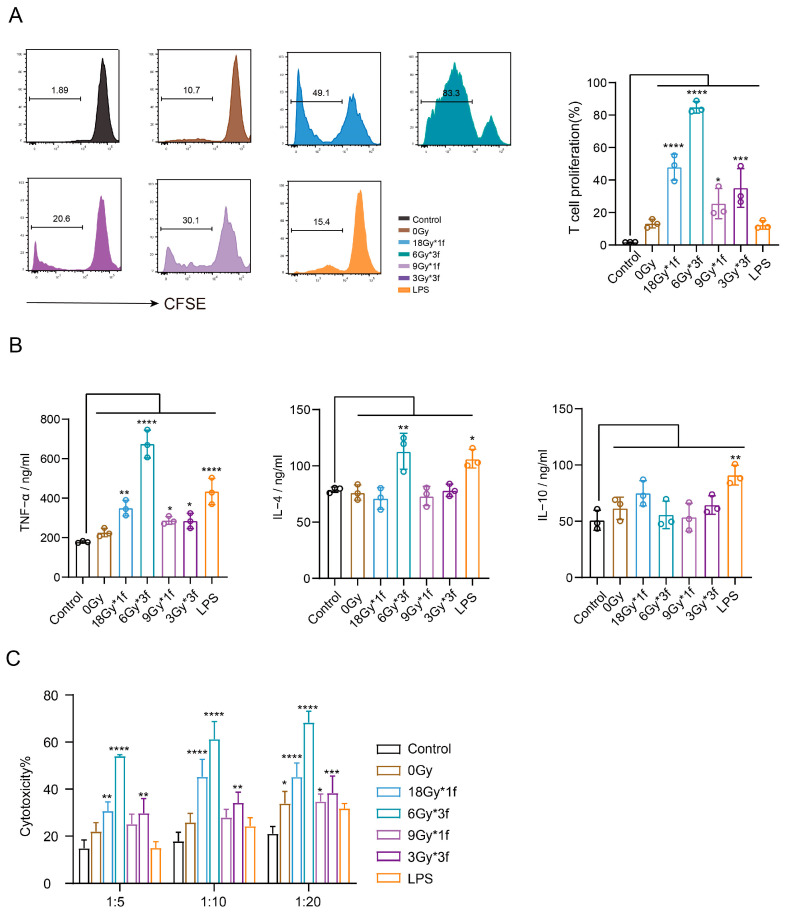

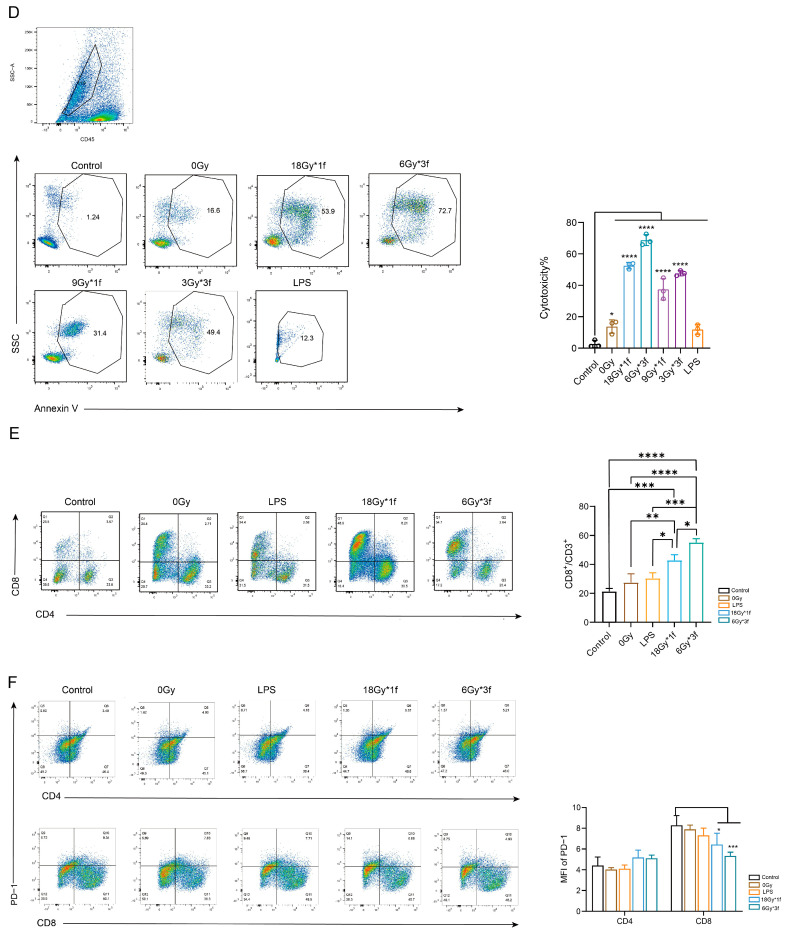
DCs loaded with RA-TEXs stimulated the proliferation and activation of T cells in vitro. (**A**). Flow cytometry was used to detect T cell proliferation after CFSE staining. (**B**). Cytokine production of TNF-α (the left), IL-4 (the middle), and IL-10 (the right) were analyzed by ELISA. (**C**). An LDH assay kit was used to detect the cytotoxicity of CTL cells stimulated by different groups of DCs. (**D**). The CD45^−^anexinV^+^ cell population was analyzed by flow cytometry to obtain the early/late apoptotic cell ratio of tumor cells from different treatments. (**E**). The proportion of CD4^+^ and CD8^+^ T cells was analyzed using flow cytometry. (**F**). The expression of PD-1 in CD3^+^CD4^+^ and CD3^+^CD8^+^ T cells was detected by flow cytometry staining. * *p* < 0.05, ** *p* < 0.01, *** *p* < 0.001, **** *p* < 0.0001.

To determine the effect of RA-TEXs on the ability of DCs to induce T lymphocyte proliferation, in vitro co-incubation experiments were performed. The study showed that the control group of DCs did not cause T cell proliferation without antigenic peptide stimulation. However, co-incubation with RA-TEX-stimulated BMDCs increased T cell proliferation, with 6Gy*3f-TEXs boosting it the most, reaching 83% (Figure 5A).

Cytokines secreted by T cells were assayed, including TNF-α, IL-10, and IL-4, associated with generating antitumor CTL responses and tumor immune surveillance [27]. We found that RA-TEXs amplified TNF-α expression to different extents, with the 6Gy*3f group demonstrating a significant, over three-fold increase in tumor-specific TNF-α. There was a significant elevation in IL-4 levels exclusively in the LPS and 6Gy*3f-TEXs group, whereas RA-TEXs did not result in a statistically significant increase in IL-10 levels compared to the control group (Figure 5B). In summary, this suggested that RA-TEXs, particularly 6Gy*3f-TEXs, potentiate T cell activation, which is likely attributable to enhanced DC maturation and antigen presentation.

Further investigation showed that RA-TEXs enhanced CTL-specific cytotoxic activity against LLC-EGFR19Del tumor cells compared to iDCs. LDH results confirmed that T cells stimulated by some TEXs-DCs exhibited stronger cytotoxic effects than the control group at three potency-target ratios. Notably, T cells stimulated by DCs-loaded with 6Gy*3f-TEXs at a 1:20 ratio had a significantly higher average cytotoxic rate, surpassing 60%, compared to iDCs and LPS groups (Figure 5C). In addition, we assessed the percentage of early or late apoptotic tumor cells (CD45^−^) in tri-cellular co-cultures using annexin V staining. Results also showed that the 6Gy*3f group had the highest CTL killing efficacy, with about 70% of tumor cells being stained by annexin V (Figure 5D).

PD-1 on T cells binding to PD-L1 on tumor cells leads to “immune escape”, inhibiting T cell activation and subsequent killing effects [28]. Thus, we represented 18Gy*1f and 6Gy*3f as RA-TEXs and iDCs, LPS, and C-TEXs as controls and the proportion of CD8^+^/CD3^+^ T cells and PD-1 expression on T cells. The study found that RA-TEXs at doses of 18Gy*1f and 6Gy*3f significantly increased (Figure 5E). Additionally, 6Gy*3f-TEXs-loaded DCs loaded with 6Gy*3f-TEXs reduced PD-1 expression in CD8^+^ T cells significantly (Figure 5F) suggests that 6Gy*3f-TEXs-loaded DCs loaded with 6Gy*3f-TEXs primarily stimulate an immune response to eliminate tumor cells through TAA-specific CTL activation and proliferation.

### 3.6. EVs Secreted by Radial Cells Enhance DC-Mediated Anti-Tumor Responses in C57BL/6 Mice WRP Avoiding Adverse Effects Such as Lungs

**Figure 6:**
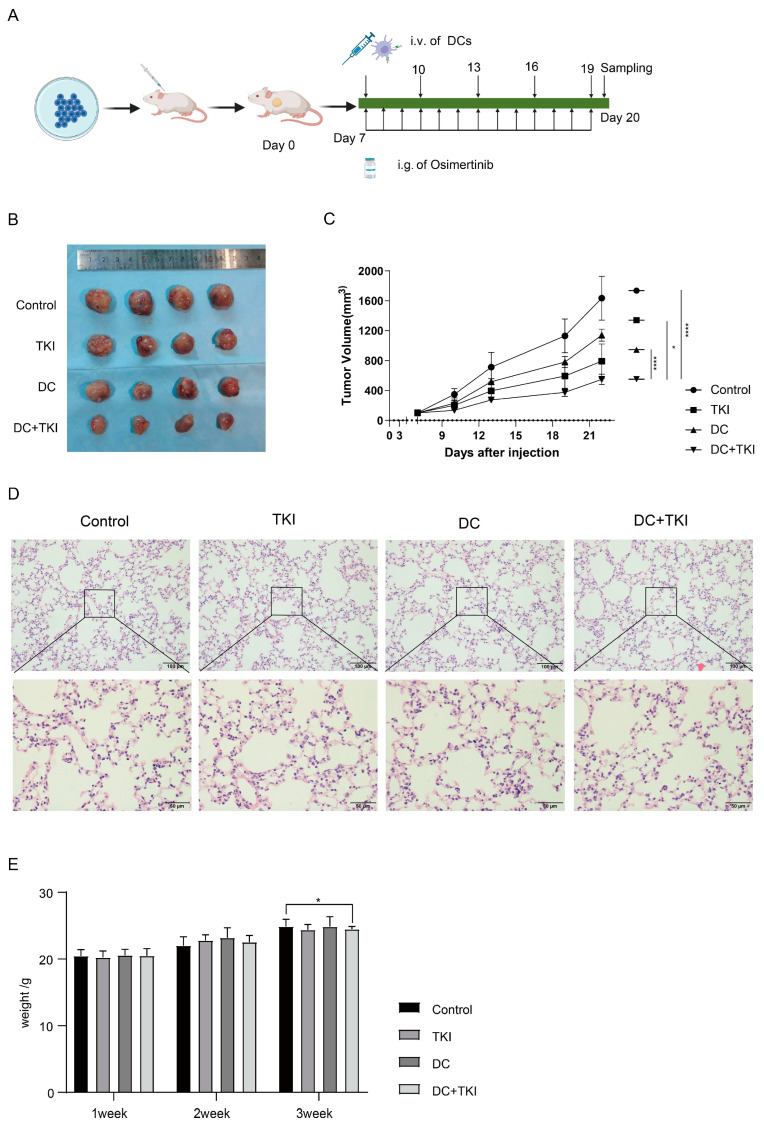

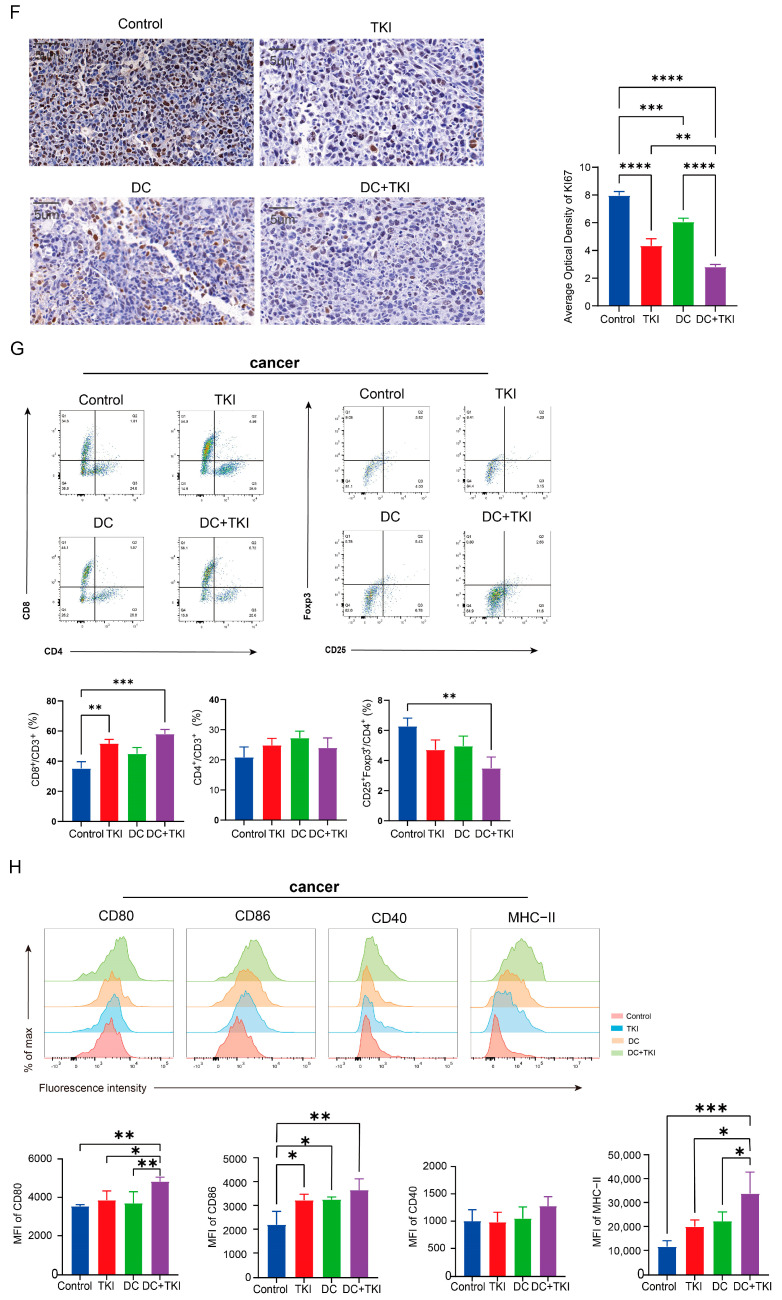

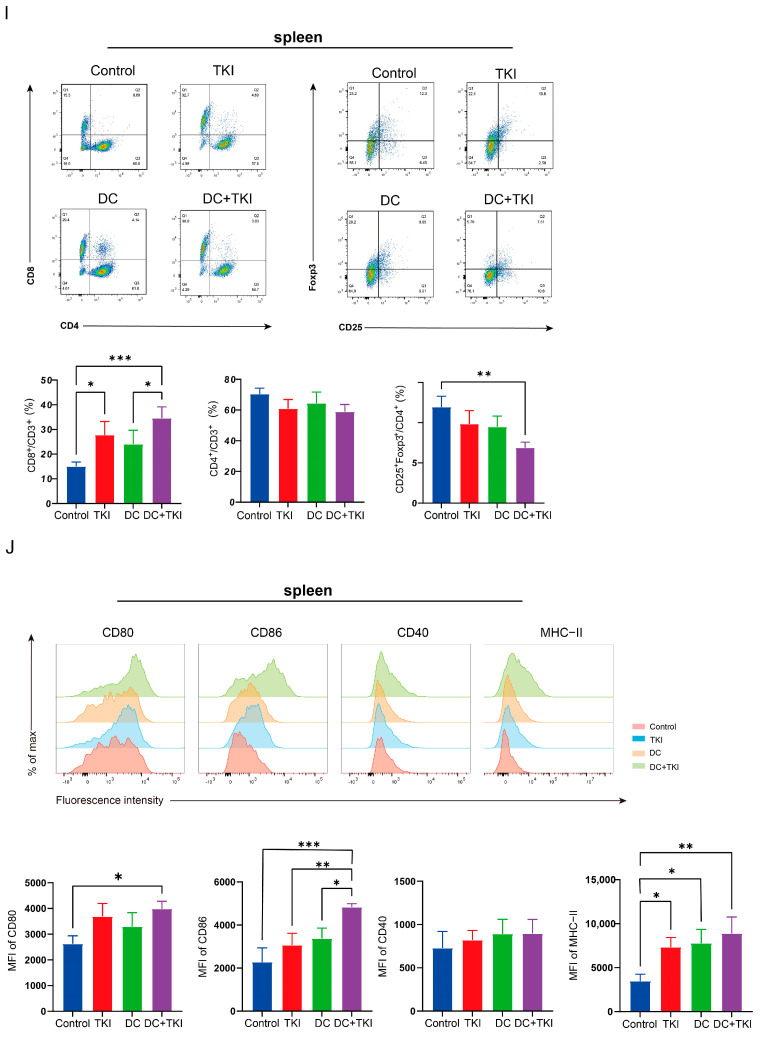
DC-loaded RA-TEXs combined with Osimertinib inhibited the growth of tumors with EGFR19Del in vivo, with an aggressive anti^−^tumor immune microenvironment and without incidence of pneumonia. (**A**). Groups of four mice were inoculated subcutaneously with 1 × 10^6^ LLC-EGFR19Del cells on day 0. On the seventh day, DCs are administered every three days via tail-vein injection. Gavage with Osimertinib was given to mice every day from day 7 to day 19. The mice were sacrificed on day 20 to evaluate the immune response in the tumor, spleen, or blood. (**B**,**C**). Photographs and volumes of tumor tissues from mice. (**D**). Sections from the lung were observed by HE staining. (**E**). Body weights of mice in each group were measured every week. (**F**). KI67 immunohistochemical staining of tumor tissues of four groups with data statistics. (**G**,**I**). The tumor or spleen of C57 mice was stained using anti-mouse CD3, CD8, CD4, CD25, or Foxp3 and analyzed using flow cytometry to detect CD4, CD8, and Treg. (**H**,**J**). DCs from the tumor or spleen were gated as CD45^+^ CD11c^+^ cells and analyzed to evaluate the expression of MHC-II, CD40, CD80, and CD86. The Schema diagram of Figure 6A was created with BioRender.com. * *p* < 0.05, ** *p* < 0.01, *** *p* < 0.001, **** *p* < 0.0001.

Research indicates that high-dose segmentation RT can expose tumor-specific antigens, creating an “in situ vaccination” effect [29]. Combining the previous data, we investigated the antitumor effects of 6Gy*3f-TEXs-stimulated DCs and TKIs in tumor-bearing mice (Figure 6A). Our findings showed that Osimertinib consistently inhibited tumor growth. However, DCs stimulated by 6Gy*3f-TEXs also caused a slight inhibition of tumors, mainly in the later stages of treatment. The combined therapy matched the effectiveness observed in earlier animal experiments, significantly reducing tumor volume compared to the control group (Figure 6B,C). Therapeutic safety was assessed. HE staining of sections showed no pathological feature in viscera, which avoided pneumonia caused by radiotherapy (Figure 6D and Figure S7). There were no significant differences in body weights among the four groups, with a steady increase being seen over two weeks (Figure 6E).

Immunohistochemical analysis of KI67 on tumor tissues showed that DCs stimulated by 6Gy*3f-TEXs, Osimertinib, and their combination significantly reduced tumor growth and malignancy compared to other treatments (Figure 6F). An immune cell assay revealed a significant increase in CD8^+^ T cell infiltration of tumor tissue in the combined treatment group and a reduction in Tregs (Figure 6G). Additionally, the expression of markers on CD11c^+^ cells within the TME indicated that mature DCs tended to accumulate in the tumor tissues of the combined treatment group (Figure S9). This was mainly shown by the increased CD80, MHC-II, and CD86 levels in DCs (Figure 6H). Blood (Figure S8) and spleen (Figure 6I) analyses of T cell populations showed consistent results. The infiltration maturation markers on DCs increased in the spleen, mainly in MHC-II and CD86 (Figure S9 and Figure 6J). Additionally, macrophage polarization in both the tumor and spleen remained unchanged by the treatment (Figures S10 and S11).

These initial findings suggest that the combination treatment effectively triggered tumor-specific CTL responses and inhibited tumor growth in vivo, with increased infiltration of mature DC and CD8^+^ T cells, the main effector cells in the local anti-tumor response.

## 4. Discussion

Over one-third of NSCLC cases are locally advanced and inoperable, usually being treated with concurrent chemoradiation, although the effectiveness of this against EGFRm NSCLC is poor [30]. EGFR-TKIs have notably improved survival rates for EGFRm-NSCLC, offering new potential for RT. For patients with stage IIIB-IV EGFRm lung adenocarcinoma treated with EGFR-TKIs, adding RT significantly improves overall survival [31]. A phase II trial showed a 96% local control rate for thoracic tumors using concurrent EGFR-TKIs and RT in advanced or metastatic NSCLC. The median progression-free survival was 10.2 months, while time to progression was 6.3 months and overall survival was 21.8 months, indicating that combination therapy is safe and effective [32]. However, medical professionals have been alerted to the risk of radiation RP in patients treated with both TKIs and TRT. In a study, 37.7% of patients with unresectable stage III NSCLC developed grade 2 or higher RP after concurrent EGFR-TKIs and RT [33]. A 2024 meta-analysis found a 3.8% chance of severe RP with this combination [34]. Our work confirmed these two points suggested by the literature. The mechanisms explaining the link between Osimertinib and RP include its role as an EGFR inhibitor, which hinders alveolar epithelial cell growth and the repair of radiation damage. Additionally, Osimertinib acts as a radiation sensitizer, reducing G2/M phase arrest and delaying DNA repair in irradiated cells, thereby increasing radiation damage in normal lung tissue [35]. Instances of lymphocytic and eosinophilic infiltration have been noted in some cases of Osimertinib-induced interstitial lung disease, posing a risk in regard to RP [36]. This highlights the need for careful consideration when combining EGFR TKIs with RT.

More and more research on combined therapy with RT can trigger radiation-induced immunogenic death, boosting immune-mediated tumor elimination in the TME. Radiation-induced cell death types include mitotic catastrophe, apoptosis, necrosis, necrotic apoptosis, senescence, and autophagy, which can trigger immunogenic responses [37]. Our research found that a high-dose segmentation pattern (6Gy*3f) led to more autophagy, apoptosis, and ICD than conventional radiation therapy and increased EV secretion. Tsai’s study revealed that human breast, prostate, and glioma tumor cells responded differently to a single (10Gy) versus fractionated (2Gy*5f) radiation dose, with IFN-related genes upregulated only in fractionated radiation cases [38]. Radiation doses over 12–18Gy activate the DNA exonuclease Trex1, reducing the immunogenicity of irradiated cells by degrading their cytoplasmic DNA and lowering Interferon Beta production [9].

Understanding EVs’ role in immune response, aiding tumor survival and progression under various conditions, is essential for creating new therapies and effective tools [39]. The exact impact of EVs from irradiated tumor cells on immune responses, whether enhancing or inhibiting anti-tumor immunity, remains under investigation, though recent studies are providing insights. A study found that, in a mouse model of breast cancer, EVs from irradiated breast cancer cells, unlike those from non-irradiated cells, triggered tumor-specific CD8^+^ T cell responses and inhibited tumor growth. These EVs delivered double-stranded DNA to DCs, enhancing co-stimulatory molecule expression and STING-dependent IFN-I activation, correlated with radiation dose [40]. In our experiment, EVs from lung adenocarcinoma cells exposed to 6Gy×3f-irradiation increased the expression of surface co-stimulatory molecules during DCs’ maturation and the secretion of pro-inflammatory cytokines, such as IFN-γ and IL-12, thereby exhibiting stronger immunogenicity. RA-TEXs enhance T lymphocyte growth and tumor cell destruction by DC cross-presentation more effectively than LPS, which lacks sufficient tumor-specific antigen messaging. Combining DCs loaded with 6Gy*3f-TEXs and Osimertinib can inhibit tumor growth and immunosuppression.

Studies show that EGFRm lung tumors generally have fewer tumor-infiltrating lymphocytes compared to wild-type tumors. Patients with EGFRm lung cancer often benefit less from immunotherapy. EGFR-19 Del mutations may suppress anti-tumor immunity by inactivating DCs via EVs, weakening the tumor microenvironment [16]. Therefore, it is important to explore whether EVs from irradiated lung cancer cells can affect immune cells and enhance anti-tumor responses in EGFRm tumors. Our study investigated the ability of DCs to stimulate T cells and compared the immunogenicity of RA-TEXs and C-TEXs, as well as the anti-tumor response of 6Gy*3f-TEXs in mice. Notably, the treatment increased CD8^+^ T lymphocyte infiltration in peripheral blood, spleen, and tumors and enhanced DC maturity, which is associated with a positive prognosis in several cancers [41].

## 5. Conclusions

In summary, our study examined the effectiveness of combining EGFR-TKIs and local radiation for EGFRm lung adenocarcinoma, focusing on pneumonitis risk. We confirmed the immunomodulatory effects of 6Gy*3f-TEXs and 6Gy*3f-TEXs-stimulated DCs combined with TKIs showed great tumor growth inhibition and better pneumonia control than RT, enhancing anti-tumor immunity in vivo and suggesting improved integration of RT with TKIs for future treatments. Our research acknowledges certain limitations. The preliminary exploration has affirmed the immunogenicity of radiation patterns and the EVs they stimulate through in vitro and in vivo experiments. Still, it does not include cases where tumors disappear entirely, possibly due to the persistence of cancer stem cells or immunosuppressive factors. Further sequencing and analysis of the EV cargo which influences DC function under radiation conditions could elucidate underlying mechanisms more comprehensively. In addition, improved delivery methods and engineered EVs may enhance the effectiveness of DC therapy, offering significant optimization opportunities.

## Data Availability

All data are available from the corresponding author upon reasonable request.

## Supplementary Materials

The following supporting information can be downloaded at: https://www.mdpi.com/article/10.3390/vaccines12121412/s1. Figure S1: Morphological changes in cells after radiation. Figure S2: H&E-stained image of internal organs in the first animal experiment. Figure S3: T cell analysis of peripheral blood in the first animal experiment. Figure S4: Proportion of DC cells detected by flow cytometry within tumors and the spleens in the first animal experiment. Figures S5 and S6: Macrophage analysis within tumors or spleens in the first animal experiment. Figure S7: H&E-stained image of Internal organs of the second-part animal experiment. Figure S8: T cell analysis of peripheral blood in the second-part animal experiment. Figure S9: Proportion of DC cells detected by flow cytometry within tumors and spleens in the second-part animal experiment. Figures S10 and S11: Macrophage analysis within tumors or spleens in the second part animal experiment.
