# Supplementary material for: Radiation-Induced Tumor-Derived Extracellular Vesicles Combined with Tyrosine Kinase Inhibitors: An Effective and Safe Therapeutic Approach for Lung Adenocarcinoma with EGFR19Del
Source: Vaccines (Basel). 2024 Dec 14;12(12):1412. doi: 10.3390/vaccines12121412 (PMC11680254; doi:10.3390/vaccines12121412)
Supplement: Supplementary file 1 [file vaccines-12-01412-s001.zip › S-3-4-5-6.pdf]

S3

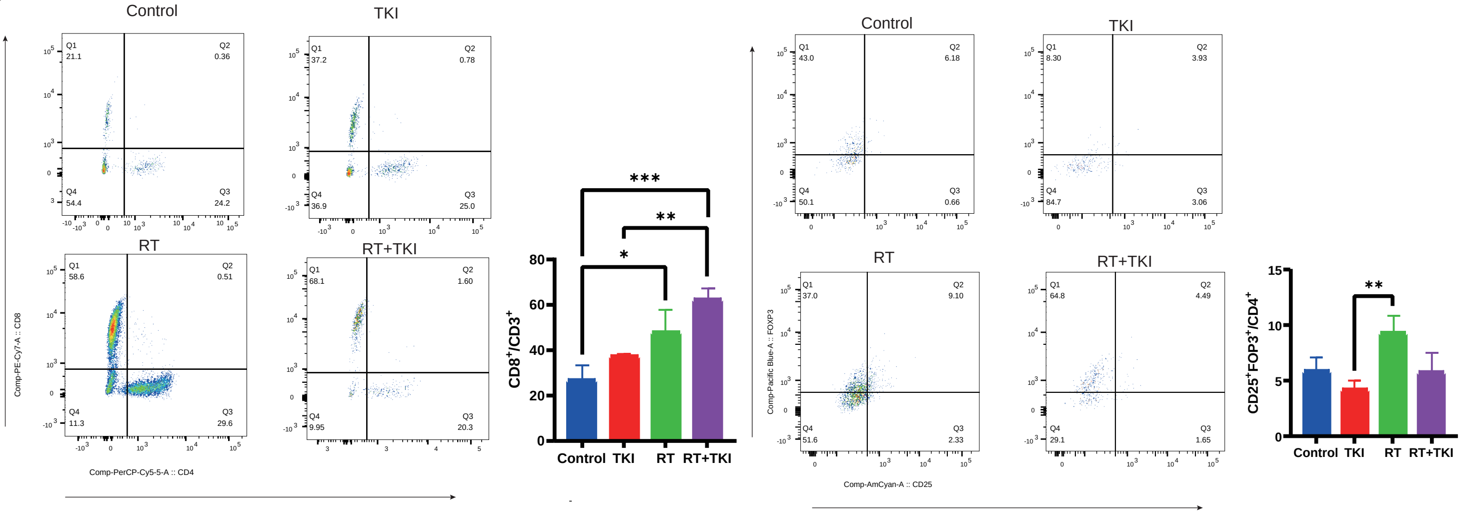

Control
TKI
RT
RT+TKI
Comp-PE-Cy7-A :: CD8
Comp-PerCP-Cy5-5-A :: CD4
CD8+/CD3+
Control TKI RT RT+TKI
Comp-Pacific Blue-A :: FOXP3
Comp-AmCyan-A :: CD25
CD25+FOP3+/CD4+


S4

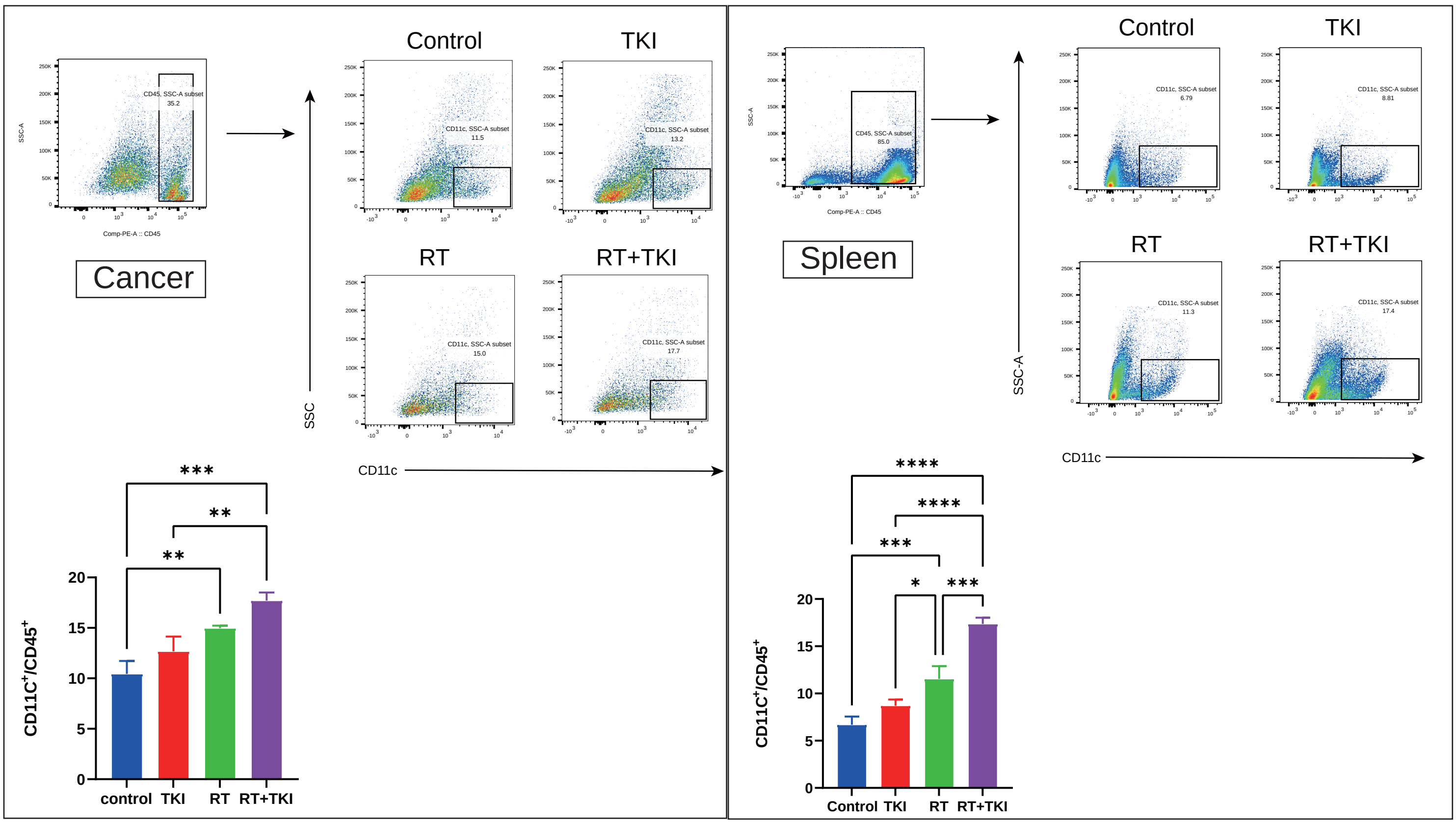

Cancer
Control
TKI
RT
RT+TKI
SSC
CD11c
CD11C+/CD45+
control TKI RT RT+TKI
Spleen
SSC-A
Control TKI RT RT+TKI

S5

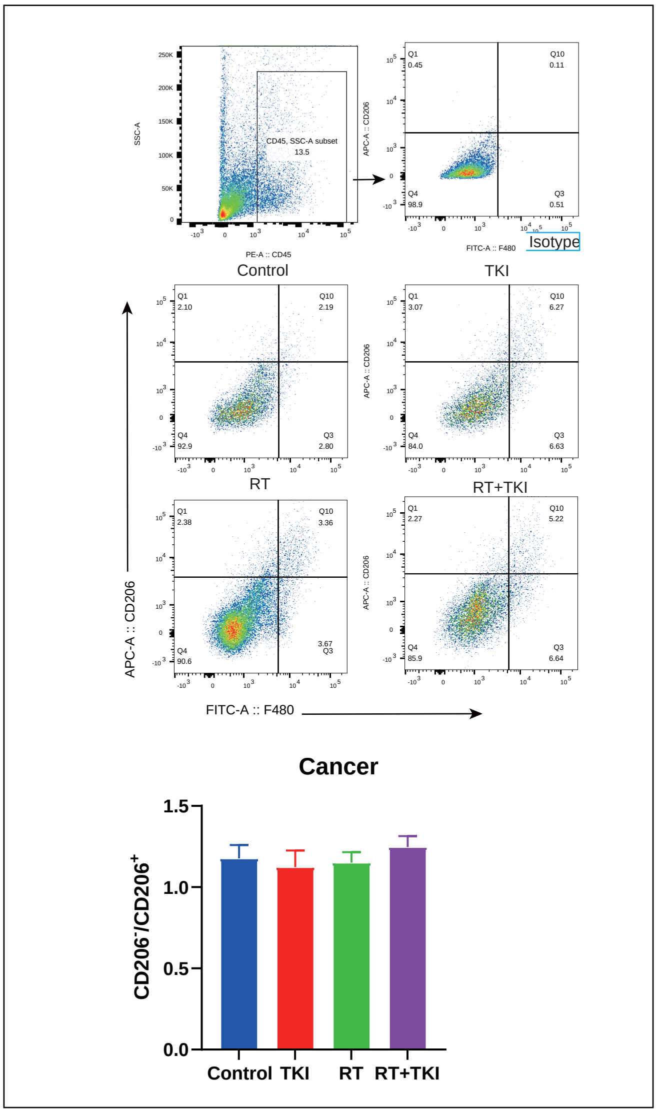


S6

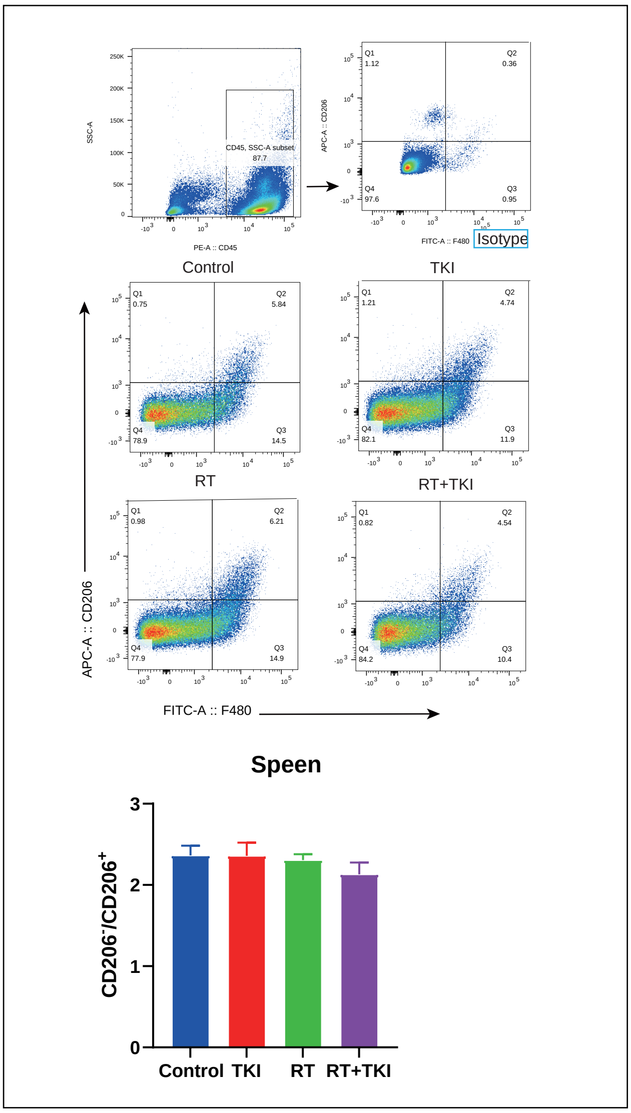


Figure S3-6: Analysis of immune cell populations in response to treatment with Control, TKI, RT, and RT+TKI. S3. Flow cytometry analysis of T cell proportions in peripheral blood at four groups, with $CD8^+$ T cells on the left and Treg on the right. S4.Proportion of DC cells detected by flow cytometry in tumor tissue(left) and in the spleen(right). S5-6. Distribution of differently polarized macrophages in the tumor (S5) and spleen (S6). * $p < 0.05$, ** $p < 0.01$, *** $p < 0.001$.
