# Supplementary material for: Radiation-Induced Tumor-Derived Extracellular Vesicles Combined with Tyrosine Kinase Inhibitors: An Effective and Safe Therapeutic Approach for Lung Adenocarcinoma with EGFR19Del
Source: Vaccines (Basel). 2024 Dec 14;12(12):1412. doi: 10.3390/vaccines12121412 (PMC11680254; doi:10.3390/vaccines12121412)
Supplement: Supplementary file 1 [file vaccines-12-01412-s001.zip › S-7.pdf]

S7

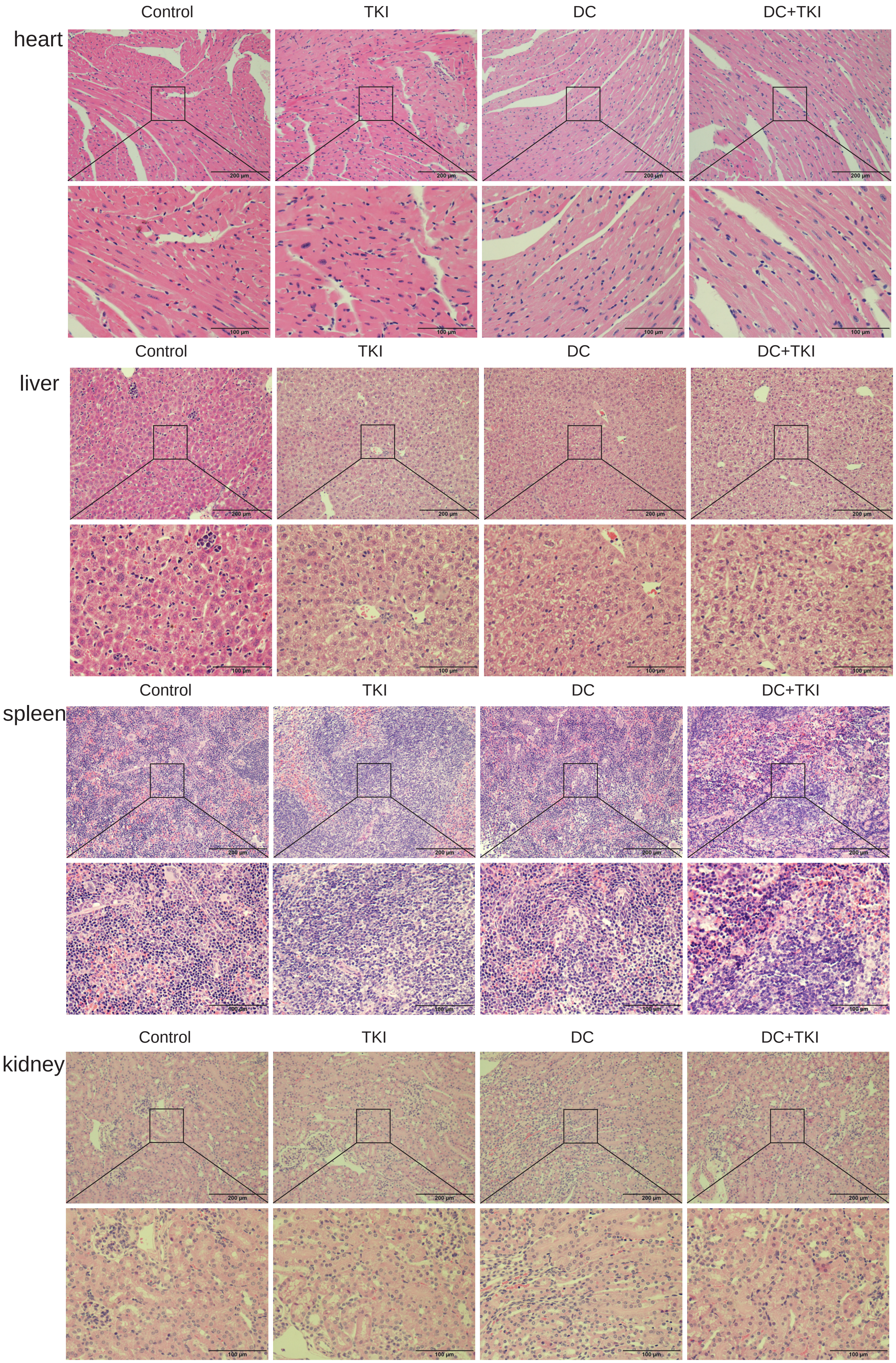


S7. The effects of different treatment (Control,TKI, DC, DC+TKI) on animal visceral organs (heart, liver, spleen, kidney). Histopathological assessment was conducted by H&E staining.
