# Supplementary figures and images for: Radiation-Induced Tumor-Derived Extracellular Vesicles Combined with Tyrosine Kinase Inhibitors: An Effective and Safe Therapeutic Approach for Lung Adenocarcinoma with EGFR19Del
Source: Vaccines (Basel). 2024 Dec 14;12(12):1412. doi: 10.3390/vaccines12121412 (PMC11680254; doi:10.3390/vaccines12121412)

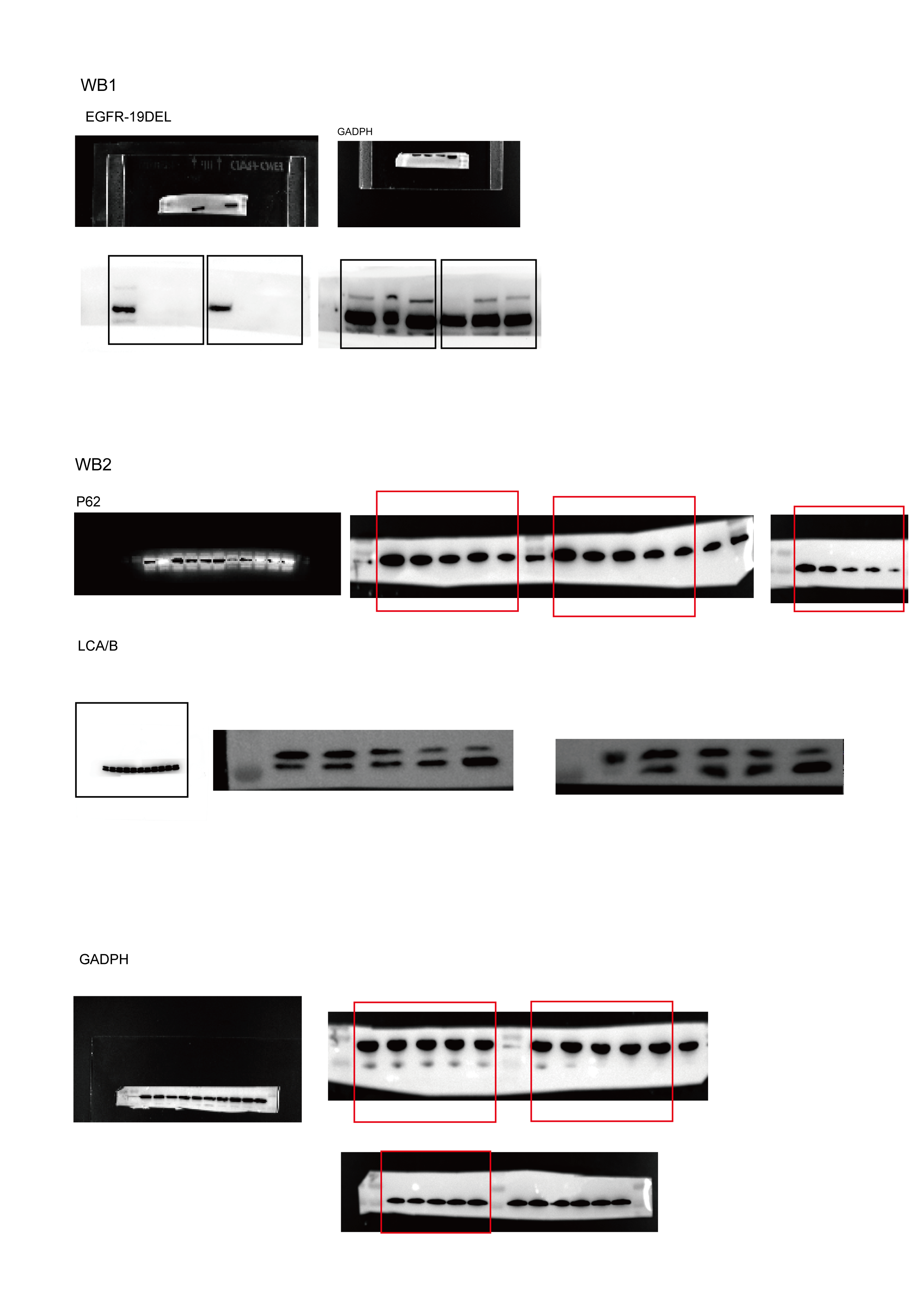

Supplement: Supplementary file 1 [file vaccines-12-01412-s001.zip › WB-data_画板 1.tif]
